# Targeted Extracellular Vesicle Gene Therapy for Modulating Alpha-Synuclein Expression in Gut and Spinal Cord

**DOI:** 10.3390/pharmaceutics15041230

**Published:** 2023-04-13

**Authors:** Maria Izco, Martin Schleef, Marco Schmeer, Estefania Carlos, Guglielmo Verona, Lydia Alvarez-Erviti

**Affiliations:** 1 Laboratory of Molecular Neurobiology, Center for Biomedical Research of La Rioja (CIBIR), Piqueras 98, 3th Floor, 26006 Logroño, Spain; 2 PlasmidFactory GmbH & Co. KG, 33607 Bielefeld, Germany; 3 Centre for Amyloidosis, UCL Medical School, Rowland Hill Street, London NW3 2PF, UK

**Keywords:** Parkinson’s disease, alpha-synuclein, gene therapy, exosomes, spinal cord, intestine

## Abstract

The development of effective disease-modifying therapies to halt Parkinson’s disease (PD) progression is required. In a subtype of PD patients, alpha-synuclein pathology may start in the enteric nervous system (ENS) or autonomic peripheral nervous system. Consequently, strategies to decrease the expression of alpha-synuclein in the ENS will be an approach to prevent PD progression at pre-clinical stages in these patients. In the present study, we aimed to assess if anti-alpha-synuclein shRNA-minicircles (MC) delivered by RVG-extracellular vesicles (RVG-EV) could downregulate alpha-synuclein expression in the intestine and spinal cord. RVG-EV containing shRNA-MC were injected intravenously in a PD mouse model, and alpha-synuclein downregulation was evaluated by qPCR and Western blot in the cord and distal intestine. Our results confirmed the downregulation of alpha-synuclein in the intestine and spinal cord of mice treated with the therapy. We demonstrated that the treatment with anti-alpha-synuclein shRNA-MC RVG-EV after the development of pathology is effective to downregulate alpha-synuclein expression in the brain as well as in the intestine and spinal cord. Moreover, we confirmed that a multidose treatment is necessary to maintain downregulation for long-term treatments. Our results support the potential use of anti-alpha-synuclein shRNA-MC RVG-EV as a therapy to delay or halt PD pathology progression.

## 1. Introduction

Although Parkinson’s disease affects 2–3 % of the population over 65 years of age [1] and its prevalence has been estimated to double over the next 30 years [2], current treatment options comprise dopamine-restoring, symptomatic therapies focused on alleviating and controlling disease motor and non-motor symptoms [3]. Even with the best medical care, this increasingly prevalent disease is devastating for the patients and has serious adverse social and economic consequences. Despite scientific advances in Parkinson’s disease research, effective treatments to alter the underlying neurodegenerative process or halt disease progression are still lacking. In the prodromal stage, most Parkinson’s disease patients developed non-motor symptoms, with the most frequent symptoms being the impairment in olfaction and the gastrointestinal dysfunction, in particular constipation [4].

The possibility of stopping Parkinson’s disease progression at early clinical or pre-clinical stages could represent a breakthrough in the treatment, as patients may not even develop motor symptoms. Unless the primary cause of Parkinson’s disease in the majority of patients is not known, current data point to a central role of alpha-synuclein in Parkinson’s disease pathology. A key pathological feature of Parkinson’s disease is the presence of alpha-synuclein aggregates, and there is increasing evidence that the transmission of pathological alpha-synuclein between neurons plays a central role in the progression and pathogenesis of Parkinson’s disease [5,6]. A recent hypothesis suggests that Parkinson’s disease subtypes are determined by the location of the initial alpha-synuclein pathology; in the brain-first subtype, the pathology may originate in the brainstem or limbic system, and in the body-first subtype the pathology may start in the enteric nervous system or autonomic peripheral nervous system [7]. The hypothesis in the body-first cases suggests that alpha-synuclein aggregates originate in the enteric nervous system and spread via the vagus nerve to the lower brainstem [8,9]. In accordance with this, alpha-synuclein pathology can be found in the large intestine of Parkinson’s disease patients up to 20 years before the diagnosis [10], and a recent study demonstrated a decreased risk of Parkinson’s disease in patients who went under truncal vagotomy [11]. Consequently, strategies to decrease the expression of alpha-synuclein in the enteric nervous system will be an attractive approach to prevent Parkinson’s disease progression at pre-clinical stages, before pathology can affect the brain and before clinical debut, in a subtype of Parkinson’s disease patients.

Gene therapy is a powerful tool to downregulate the expression of alpha-synuclein and a promising approach for the treatment of Parkinson’s disease. A major challenge for clinical applications is the development of vehicles for delivery to the central nervous system that cross the blood-brain barrier and deliver the molecules specifically into the central nervous system following peripheral administration, avoiding the activation of the immune response and allowing repeat administration.

We have designed and generated a new delivery approach using modified extracellular vesicles (EV), which specifically target the central nervous system, by placing the rabies virus glycoprotein peptide (RVG peptide) in the external surface of the EV [12]. In a recent study, we demonstrated the potential of intravenous administration of RVG-targeted EV (RVG-EV) to deliver shRNA minicircles (shRNA-MC) to the brain. The treatment induces long-term alpha-synuclein downregulation in the brain and prevents dopaminergic cell death and motor abnormalities in a Parkinson’s disease mouse model [13]. These results confirmed the potential of shRNA-MC delivered by RVG-EV to induce long-term downregulation of protein expression in the brain after systemic injection and halt the pathological progression. Our approach has the advantage that it is minimally invasive and reduces the protein synthesis or post-translational modification preventing its aggregation [12]. However, this new therapeutic approach for long-term Parkinson’s disease treatment requires further characterization.

In the present study, we aimed to assess if anti-alpha-synuclein shRNA-MC RVG-EV could downregulate alpha-synuclein expression in organs affected in pre-clinical stages of Parkinson’s disease, as intestine and spinal cord. Moreover, we investigated if our therapy could halt the disease progression after the development of alpha-synuclein pathology, and we evaluated the duration of the therapeutic effect. Our results confirmed the downregulation of alpha-synuclein mRNA and protein levels in the intestine and spinal cord of mice treated with anti-alpha-synuclein shRNA-MC RVG-EV. The treatment with RVG-EV loaded with anti-alpha-synuclein shRNA-MC after the appearance of pathology was effective to downregulate alpha-synuclein expression not only in the brain, but also in the intestine and spinal cord. Finally, we demonstrated that a multidose treatment is necessary to maintain the alpha-synuclein downregulation for long-term treatments.

## 2. Materials and Methods

### 2.1. Animals

Normal male C57BL6/C3H F1 mice (8 to 9 weeks old) were purchased from Charles River Laboratories. Animals were housed under environmentally controlled standard conditions with a 12-h light/dark cycle and provided with food and water ad libitum. All procedures involving animals were carried out in accordance with the European Communities Council Directive (2010/63/UE) and Spanish legislation (RD53/2013) on animal experiments and with approval from the ethical committee on animal welfare for our institution (Órgano Encargado del Bienestar Animal del Centro de Investigación Biomédica de La Rioja, OEBA-CIBIR). All efforts were made to minimize suffering and pain of the animals.

### 2.2. Study Design

Mice received an injection of sonicated murine alpha-synuclein pre-formed fibrils (PFFs) into the dorsal striatum as described below. In one study, mice received two intravenous injections of RVG-EV containing alpha-synuclein shRNA-MC or vehicle (glucose 5%) 2 and 45 days after alpha-synuclein PFF intrastriatal injection and were sacrificed 90 days post injection (dpi). In compliance with the 3Rs for refining, reducing, and replacing animals for research purposes, we obtained the spinal cord and intestine samples of the present study from a previous published study [13]. In another study, mice received two doses of alpha-synuclein shRNA-MC loaded into RVG-EV or vehicle (glucose 5%) 35 and 80 days after alpha-synuclein PFF injection, while another group received only one dose of alpha-synuclein shRNA-MC RVG-EV 35 days after PFF injection, and mice were sacrificed 140 dpi. Control animals in both studies were injected into the striatum with an equal volume of sterile PBS. Brains, spinal cords, and intestines were removed and immediately frozen and stored at −80 °C until used for biochemical analysis. For histological studies, spinal cords and intestines were removed after transcardial perfusion.

### 2.3. Preparation of Mouse Wild-Type Alpha-Synuclein Preformed Fibrils

Mouse wild-type alpha-synuclein PFFs were prepared in sterile PBS (pH 7.4) from alpha-synuclein monomer 5 mg/mL by agitation (250 rpm at 37 °C) for one week. Fibrils were isolated by centrifugation at 10.600× *g* for 15 min. The total amount of fibrils formed was determined using a Jasco V-650 spectrophotometer by the difference between the total amount of protein in solution before incubation and the total amount of protein left in the solution in the supernatant at the end of the incubation period. The pellet was then suspended at a concentration of 1 mg/mL in sterile PBS. Fibril formation was confirmed by Congo red staining. On the day of the surgery, alpha-synuclein PFFs were sonicated for two cycles of 6 s 50 % power (10 microns amplitude) using a probe sonicator (Soniprep 150, MSE) to generate the alpha-synuclein seeds. A new aliquot of sonicated alpha-synuclein PFF was prepared every day of surgery.

### 2.4. Stereotaxic Surgery

C57BL6/C3H F1 mice were anesthetized with isoflurane and placed in a stereotaxic frame with ear bars. Mice received two unilateral 2.5 μL injections of sonicated murine alpha-synuclein PFF (5 μg in 5 μL total) into the right striatum (coordinates: AP, +0.2 mm relative to the bregma, ML, −2.0 mm relative to bregma, DV, −3.4 mm and −2.6 mm below the skull) at the rate of 0.25 μL/min as previously described [13]. Control animals were injected with an equal volume of sterile PBS.

### 2.5. shRNA-Minicircle Generation

The production of shRNA-MC is carried out in 2 major steps: the cultivation in a bioreactor and the purification by specific chromatographic steps. The cultivations were carried out at 37 °C in a MBR bioreactor (MBR BIO REACTOR, Recherswil, Switzerland) with 5 L, pH adjusted to 7.0 with 2 M sodium hydroxide solution and 2 M phosphoric acid. The oxygen concentration of 60% was controlled by varying the stirrer speed. LB-medium was used without addition of antibiotics. The bioreactor was inoculated with 50 mL of an E. coli K12 culture transformed with the parental plasmid (PP) and grown in LB-medium for approximately 15 h. The recombinase expression was induced at an OD600 » 4 by adding L-arabinose. After 1 h of further growth, cells were harvested by centrifugation, frozen, and purified by the PlasmidFactory contract manufacturing service (Bielefeld, Germany).

After successful recombination, the shRNA-MC was separated from the miniplasmid (MP). This was done by a series of chromatography steps, including an affinity chromatography step separating MP and shRNA-MC. The recombination product (shRNA-MC and MP) was further purified by affinity chromatography as previously described [14]. The sequence specific DNA binding was optimized with different ionic strength and pH values and resulted in a highly purified supercoiled monomeric shRNA-MC product.

### 2.6. Dendritic Cell Culture and RVG-EV Isolation

Primary mouse dendritic cells were harvested from murine bone marrow and cultured in complete DMEM medium supplemented with 10 ng/mL murine GM-CSF (Peprotech EC Ltd., London, UK). Following transfection of the dendritic cells with mouse RVG-Lamp2b plasmid, the cell culture medium was changed on Day 7. EV were isolated from cell culture supernatant harvested 24 h after, by serial centrifugation at 12,000× *g* for 30 min, and at 120,000× *g* for 1 h to pellet EV. EV were resuspended in 0.1 M ammonium acetate with a 27G syringe.

### 2.7. RVG-EV Treatment of Mice

A total of 150 μg of shRNA-MCs and 150 μg of RVG-EV were electroporated (450 V, 100 mA) in 5 mL electroporation buffer (1.15 mM potassium phosphate pH 7.2, 25 mM potassium chloride, 21% OptiPrep) and treated with 100 U DNase (Promega, Madison, WI, USA) at 37 °C for 30 min. After ultracentrifugation at 120,000× *g* for 1 h, RVG-EV (150 μg) were resuspended in 100 μL 5% glucose immediately before tail vein injection.

### 2.8. Western Blot Analysis

Brain and spinal cord samples were homogenized in buffer containing 10 mM Tris/HCl (pH 7.4), 0.1% SDS, protease inhibitor mixture (Thermo Scientific, Walthman, MA, USA), and Dnase (Promega). Distal intestine samples were homogenized in the same buffer containing 8 M urea. Proteins levels were determined by the Pierce BCA protein assay (Pierce BCA protein assay kit, Thermo Scientific) using bovine serum albumin as the standard. Samples (20 μg of protein) were solubilized in LDS buffer and reducing agent, separated on NuPAGE Novex 4–12% Bis-Tris Gels (Invitrogen, Walthman, MA, USA), transferred to PVDF membrane, and analyzed by western blot as previously described [13] using anti-alpha-synuclein (Abcam, Cambridge, UK, ref# ab1903, dilution 1:2000) and anti-beta-actin (Abcam, ref# ab6276, dilution 1:30,000) antibodies. Horseradish-peroxidase-conjugated anti-mouse immunoglobulin G (IgG) secondary antibody (Agilent Dako, Santa Clara, CA, USA) was detected using ECL Western Blot Substrate (Pierce, Walthman, MA, USA) and ChemiDoc MP Imaging System (Bio-Rad, Hercules, CA, USA). Signals in the linear range were quantified using ImageJ and normalized to beta-actin levels.

### 2.9. Quantitative PCR

Total RNA was isolated from frozen brain, spinal cord, and distal intestine samples using the RNeasy kit (Qiagen, Gilden, Germany) according to the manufacturer’s protocol. Subsequently, reverse transcription was performed with High -Capacity cDNA Reverse Transcription kit (Applied Biosystems, Walthman, MA, USA) as per manufacturer’s instruction. qPCR experiments were performed on a QuantStudio 5 Real-Time PCR system (Applied Biosystems) using NZY Supreme qPCR Green Mastermix (Nzytech, Lisbon, Portugal). Values were calculated using the standard ΔΔCt method. The primer sequences for alpha-synuclein (forward: 5′-GCCAAGGAGGGAGTTGTGGCTGC-3′; reverse: 5′-CTGTTGCCACACCATGCACCACTCC-3′) were synthesized by Sigma (San Luis, CA, USA) and the sequence for mouse actin (forward: 5′-TCTACAATGAGCTGCGTGTG-3′; reverse: 5′-GGTGAGGATCTTCATGAGGT-3′) was synthesized by Primer Design (Chandler’s Ford, UK).

### 2.10. Immunohistochemistry and Immunofluorescence

Mice were transcardially perfused with PBS followed by 4% paraformaldehyde (PFA) in PBS; the cords and intestines were post-fixed in 4% PFA, cryoprotected in 30% sucrose, rapidly frozen, and stored at −80 °C until use. Then, 30 µm thick coronal sections were prepared using a freezing microtome. Cord and intestine sections were washed with TBS and incubated with 3% hydrogen peroxidase to inactivate the endogenous peroxidase. After wash steps, sections were incubated with a blocking solution containing 5% normal goat serum followed by incubation overnight at 4 °C with the primary antibodies: anti-alpha-synuclein (Abcam, ref# ab1903, dilution 1:5000) and anti-phospho S129-alpha-synuclein (Abcam, ref# ab51253, dilution 1:2000). Sections were rinsed and then incubated with fluorescent or biotinylated secondary antibody of the appropriate species. All the samples were processed simultaneously to allow comparison.

### 2.11. Statistical Analysis

All data are presented as mean values ± the standard error of the mean (SEM). Statistical analyses of the data were carried out using SPSS program (version 25.0). Statistical comparisons between experimental groups were performed with the parametric one-way ANOVA followed by the Tukey HSD as indicated. When variables were non-normally distributed, statistical differences were analyzed by non-parametric Kruskal–Wallis followed by the Mann–Whitney U-test. A probability level ≥ 0.05 was considered to be statistically significant.

## 3. Results

### 3.1. Alpha-Synuclein shRNA-MC RVG-EV Therapy Downregulate Alpha-Synuclein in Spinal Cord and Intestine of Alpha-Synuclein PFF Mouse Model of Parkisnon’s Disease

**Figure 1:**
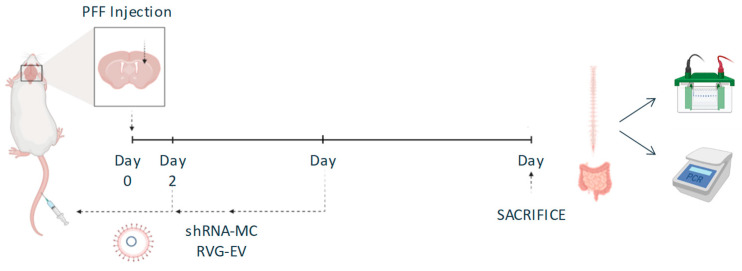
Experimental design of the study to evaluate the effect of anti-alpha-synuclein shRNA-MC RVG-EV therapy administrated intravenously.

The efficacy of anti-alpha-synuclein shRNA-MC delivered by RVG-EV to downregulate alpha-synuclein in spinal cord and intestine was evaluated using a progressive alpha-synucleinopathy mouse model based on the injection of alpha-synuclein PFFs into the striatum of normal mice. Mice received IV injections of 150 µg RVG-EV loaded with 150 µg anti alpha-synuclein shRNA-MC (*n* = 18) or IV injections of vehicle (glucose 5%; *n* = 18) 2 and 45 days after alpha-synuclein PFF or PBS (control group) injection and were sacrificed 90 dpi (Figure 1).

**Figure 2:**
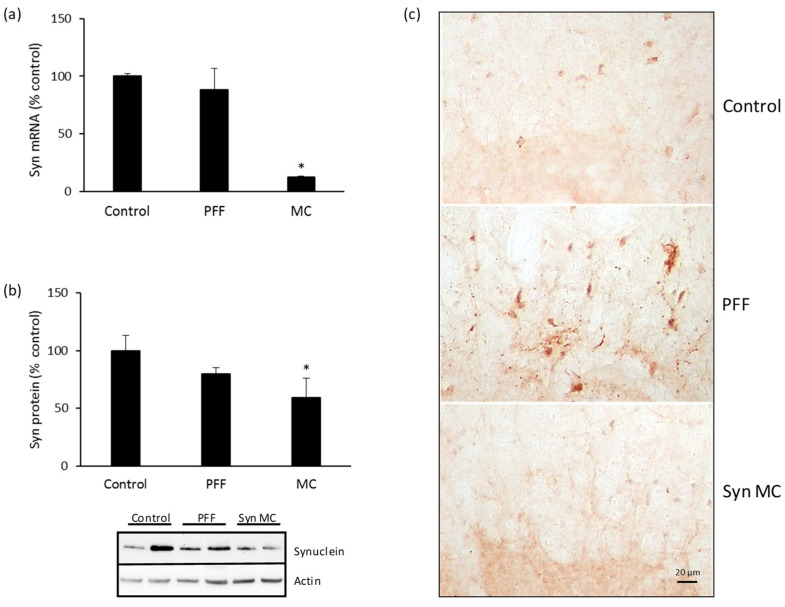
Alpha-synuclein downregulation in spinal cord of mice treated with anti-alpha-synuclein shRNA-MC RVG-EV. Analyses of alpha-synuclein mRNA expression (**a**) and protein levels (**b**) normalized to actin. Typical western blot is shown. (**c**) Cord sections were stained for S129 phospho-synuclein, typical immunohistochemical images are shown. Scale bar represents 20 µm. Values are expressed as mean ± SEM (*n* = 5). * *p* < 0.05, non-parametric Kruskal-Wallis test.

**Figure 3:**
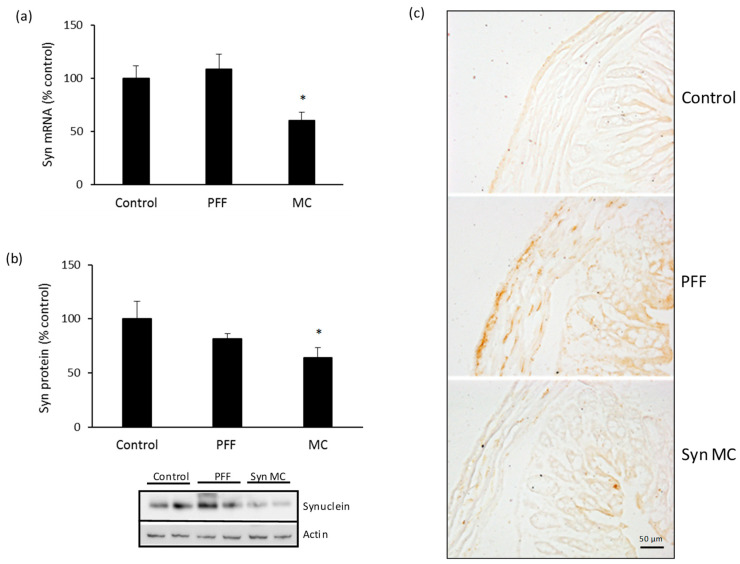
Alpha-synuclein downregulation in intestine of mice treated with anti-alpha-synuclein shRNA-MC RVG-EV. Analyses of alpha-synuclein mRNA expression (**a**) and protein levels (**b**) normalized to actin. Typical western blot is shown. (**c**) Intestine sections were stained for S129 phospho-synuclein, typical immunohistochemical images are shown. Scale bar represents 50 µm. Values are expressed as mean ± SEM (*n* = 5). * *p* < 0.05, non-parametric Kruskal–Wallis test.

After 90 days, there was a significant decrease in the mRNA levels of alpha-synuclein in the spinal cord of mice treated with anti-alpha-synuclein shRNA-MC RVG-EV (decreased 83% compared to controls) (Figure 2a). The mRNA decrease was associated with lower levels of alpha-synuclein protein (decreased 41% compared to controls) (Figure 2b). The IV injection of anti-alpha-synuclein shRNA-MC RVG-EV also decreased mRNA levels of alpha-synuclein in the intestine of mice treated with the therapy (decreased 40% compared to controls) (Figure 3a). A concomitant decrease of alpha-synuclein protein was demonstrated in intestinal samples (decreased 37% compared to controls) (Figure 3b). The injection of alpha-synuclein PFFs into the striatum was associated with S129 phospho-alpha-synuclein-positive inclusions in the cord and intestine, the treatment with anti-alpha-synuclein shRNA-MC RVG-EV prevented the S129 phospho-alpha-synuclein aggregate formation in those tissues (Figure 2c and Figure 3c).

### 3.2. Evaluation of Alpha-Synuclein Downregulation by shRNA-MC RVG-EV Administrated after the Development of Alpha-Synuclein Pathology

**Figure 4:**
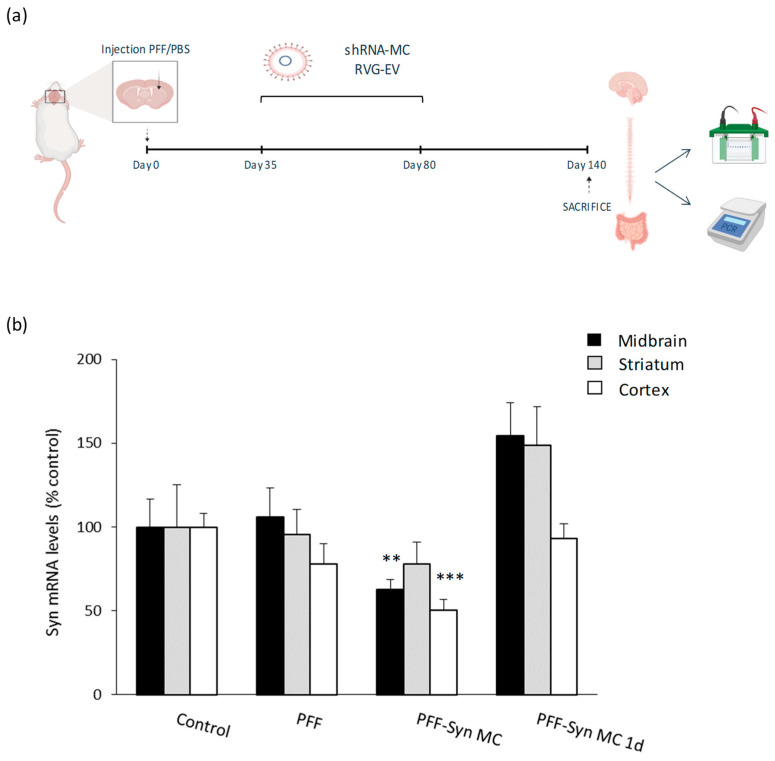
Effect of anti-alpha-synuclein shRNA-MC RVG-EV therapy administrated after the development of alpha-synuclein pathology. (**a**) Experimental design of the study is shown (**b**) Alpha-synuclein mRNA expression levels in the midbrain, striatum, and cortex of mice treated with the therapy after the development of pathology. Values are expressed as mean ± SEM (*n* = 8). ** *p* < 0.01; *** *p* < 0.001, parametric one-way ANOVA test.

By the time of the diagnosis, Parkinson’s disease patients already present massive dopaminergic neuronal loss and alpha-synuclein pathology. In order to evaluate if the anti-alpha-synuclein shRNA-MC RVG-EV therapy could be able to downregulate alpha-synuclein expression after the development of alpha-synuclein pathology, mice were treated IV with RVG-EV loaded with anti-alpha-synuclein shRNA-MC 35 and 80 days after alpha-synuclein PFF injection and were sacrificed 140 dpi. Moreover, one group of animals received only one dose of alpha-synuclein shRNA-MC RVG-EV 35 days after PFF injection in order to evaluate the duration of the therapy effect (Figure 4a).

In brain samples, we confirmed a decrease in the mRNA levels of alpha-synuclein in the midbrain (decreased 38% compared to controls), striatum (decreased 22% compared to controls, non-significant), and cortex (decreased 50% compared to controls) of mice treated with anti-alpha-synuclein shRNA-MC RVG-EV (Figure 4b). However, these levels were unaltered 105 days after the single administration of the therapy in the same brain regions (Figure 4b).

**Figure 5:**
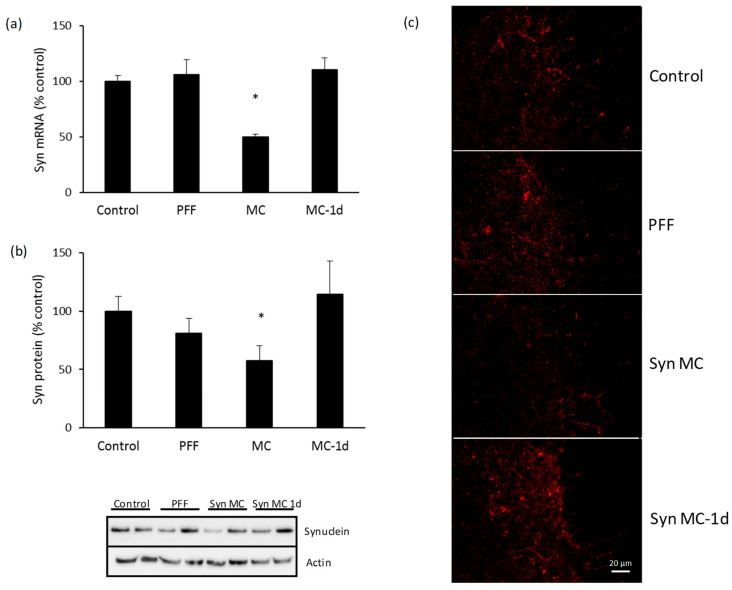
Alpha-synuclein downregulation in spinal cord of mice treated with anti-alpha-synuclein shRNA-MC RVG-EV after the development of alpha-synuclein pathology. Analyses of alpha-synuclein mRNA expression (**a**) and protein levels (**b**) normalized to actin. Typical western blot is shown. (**c**) Immunofluorescence images of total alpha-synuclein in cord sections. Scale bar represents 20 µm. Values are expressed as mean ± SEM (*n* = 5). * p < 0.05, non-parametric Kruskal–Wallis test.

**Figure 6:**
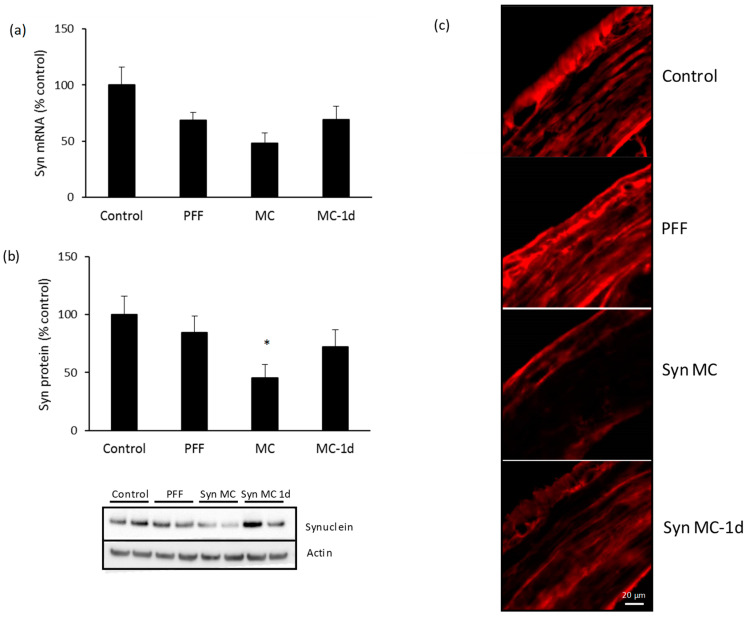
Alpha-synuclein downregulation in intestine of mice treated with anti-alpha-synuclein shRNA-MC RVG-EV after the development of alpha-synuclein pathology. Analyses of alpha-synuclein mRNA expression (**a**) and protein levels (**b**) normalized to actin. Typical western blot is shown. (**c**) Immunofluorescence images of total alpha-synuclein in intestine sections. Scale bar represents 20 µm. Values are expressed as mean ± SEM (*n* = 5). * *p* < 0.05, non-parametric Kruskal–Wallis test.

The treatment with anti-alpha-synuclein shRNA-MC RVG-EV after the development of alpha-synuclein pathology significantly reduced the mRNA levels of alpha-synuclein in the spinal cord (decreased 50% compared to controls) (Figure 5a). The mRNA downregulation was associated with decreased levels of alpha-synuclein protein (decreased 43% compared to controls) (Figure 5b,c). The treatment with only one dose of the therapy was not sufficient to reduce mRNA or protein levels of alpha-synuclein 105 days after therapy administration (Figure 5a–c). Similar results were observed in intestinal samples, with an mRNA decrease of 32% compared to controls (Figure 6a). There was a concomitant reduction of alpha-synuclein protein levels (decreased 55% compared to controls) (Figure 6b,c) in mice treated with both doses of anti-alpha-synuclein shRNA-MC RVG-EV therapy. The mRNA levels and alpha-synuclein protein levels were unaffected in mice treated with only one dose of anti-alpha-synuclein shRNA-MC RVG-EV (Figure 6a–c).

## 4. Discussion

Although current Parkinson’s disease treatments can improve clinical symptoms, there is no effective treatment options capable to alter or halt the progression of Parkinson’s disease pathology. Given the increasing evidence that the transmission of pathological alpha-synuclein between neurons plays a central role in Parkinson’s disease progression [5,6] and that pathology may start in the ENS and spreads to the brain via the vagus nerve [8,9], strategies targeting these organs could be a potential therapeutic approach to prevent progression at the prodromal stage in body-first Parkinson’s disease patients. This is the first study targeting alpha-synuclein in the intestine and spinal cord (central nervous system), supporting the potential use of anti-alpha-synuclein shRNA-MC RVG-EV as a therapy to delay or halt Parkinson’s disease pathology progression. To date, all the studies were focused in interfere with pathological alpha-synuclein dissemination by immunization or decrease alpha-synuclein level in brain and thereby halt the spread in the brain.

In a previous study, we developed and validated a new therapy using shRNA-MC delivered by RVG-EV [13]. However, the development of this new technology for long-term targeted gene downregulation in Parkinson’s disease required a detailed characterization including other organs targeted and the duration of the effect. Our study confirmed that anti alpha-synuclein shRNA-MC RVG-EV therapy decreases the total levels of alpha-synuclein protein in intestine and spinal cord in a similar range of effect previously described in different brain areas [13].

Several studies of EV biodistribution reported the presence of untargeted EV isolated from different cell types in the gastrointestinal system after iv injection [15,16]. However, the potential of untargeted or targeted EV to deliver siRNAs or shRNA into the intestine and spinal cord, and downregulate protein expression was not previously assessed. Our study demonstrated for first time that RVG-EV could efficiently deliver shRNA-MC in both tissues modulating the levels of the targeted protein.

Previous studies have highlighted the use of gene therapy for spinal cord repair and demonstrated the therapeutic potential in vivo for the treatment of spinal cord injury [17]. All these studies used viral vectors except for one that used liposomes [18], and administrated gene therapy molecules locally by intralesional, intraspinal, or intrathecal injections. There are also numerous studies that have demonstrated local gene expression of therapeutic molecules in the intestine for the treatment of inflammatory bowel diseases [19]. The majority of the studies used viral vectors, although other non-viral vectors, as nanoparticles [20,21] or liposomes [22], were also assessed successfully. The genetic material was administrated by different routes including intraperitoneal [23,24], intravenous [25,26], oral [27,28], or local [29,30,31] administration. Compared with those approaches, our therapy has the advantage that it not only targets the intestine, but also targets the spinal cord and brain, important organs affected by the alpha-synuclein pathology in Parkinson’s disease. The delivery to the enteric nervous system and central nervous system at the same time is a clear advantage and simplifies the Parkinson’s disease treatment.

Moreover, it has been demonstrated that RVG-EV intravenously injected avoids the delivery of the genetic cargo into other organs including the liver, heart, muscle, and spleen [12]. Another major challenge for clinical applications is the development of vehicles that avoid activation of the immune response, which could both preclude repeat dosing and also exacerbate the inflammation, which is an important aspect of neurodegenerative diseases. None of the current approaches, including viral vectors, in vivo transfection reagents, or liposomes, comply with the requirements. However, we have demonstrated in previous studies that the RVG-EV delivery system allows repeated diffuse delivery into the brain [12,13] without immune activation, a clear advantage for long term treatment.

shRNA-MC allows sustained episomal transgene expression for several weeks; however, as a chronic disease, the treatment for Parkinson’s disease must be effective in the long term. The chronic gene downregulation in the brain and enteric nervous system using a shRNA-MC requires repeated doses; however, previously the duration of the effect was only evaluated up to 8 weeks [13,32]. In this study, we assessed the alpha-synuclein mRNA expression 60 days and 105 days after the last shRNA-MC RVG-EV injection. The results demonstrated that 60 days after injection the mRNA level is downregulated, however, 105 days after injection the mRNA level was normal. Our results demonstrated that a multidose treatment every 2 months is necessary to maintain alpha-synuclein downregulation for long term treatments.

A limitation of the initial study was that mice were treated with the shRNA-MC RVG-exosomes therapy before the development of alpha-synuclein pathology; however, Parkinson’s disease diagnosis is based on the presence of classical motor symptoms, and at this point significant disease pathology and Parkinson’s disease progression already exist [33]. Our results confirmed that the treatment with anti-alpha-synuclein shRNA-MC RVG-EV after the development of pathology is effective to downregulate alpha-synuclein expression in the brain as well as in the intestine and spinal cord.

The results of the study suggested that shRNA-MC RVG-EV therapy could not only decrease alpha-synuclein aggregation in brain but also could reduce the formation of alpha-synuclein fibrils/aggregates in organs affected in pre-clinical stages of Parkinson’s disease. These data should be confirmed using a progressive model recently developed based in the injection of mouse alpha-synuclein PFF into the intestine of normal C57BL/6J mice [9]. The prevention of alpha-synuclein aggregation in brain and the dopaminergic degeneration in this model may suggest that this therapy could alter the course of Parkinson’s disease progression in a subset of patients.

## 5. Conclusions

Our results confirmed that shRNA-MC RVG-EV therapy reduce alpha-synuclein expression and protein levels in the spinal cord and intestine. Moreover, the treatment with RVG-EV loaded with alpha-synuclein shRNA-MC after the appearance of pathology is also effective to downregulate alpha-synuclein expression in the brain as well as in the spinal cord and intestine. Finally, a multidose treatment is necessary to maintain the alpha-synuclein downregulation for long-term treatments.

The results of this study highlight the therapeutic potential of anti-alpha-synuclein shRNA-MC RVG-EV as a therapy to delay or halt Parkinson’s disease pathology progression at pre-clinical stages.

## Data Availability

All primary data and materials will be made available under request.
